# Nanoclay/Polymer-Based Hydrogels and Enzyme-Loaded Nanostructures for Wound Healing Applications

**DOI:** 10.3390/gels7020059

**Published:** 2021-05-14

**Authors:** Angel M. Villalba-Rodríguez, Sara Martínez-González, Juan Eduardo Sosa-Hernández, Roberto Parra-Saldívar, Muhammad Bilal, Hafiz M. N. Iqbal

**Affiliations:** 1 Tecnologico de Monterrey, School of Engineering and Sciences, Monterrey 64849, Mexico; angel_villalba_r@hotmail.com (A.M.V.-R.); A01166376@itesm.mx (S.M.-G.); eduardo.sosa@tec.mx (J.E.S.-H.); r.parra@tec.mx (R.P.-S.); 2 School of Life Science and Food Engineering, Huaiyin Institute of Technology, Huaian 223003, China

**Keywords:** polymeric materials, chitosan, laponite, nanoparticles, hydrogels, biomedical, wound healing

## Abstract

Multi-polymeric nanocomposite hydrogels with multi-functional characteristics have been engineered with high interest around the globe. The ease in fine tunability with maintained compliance makes an array of nanocomposite biomaterials outstanding candidates for the biomedical sector of the modern world. In this context, the present work intends to tackle the necessity of alternatives for the treatment of diabetic foot ulcers through the formulation of nanoclay and/or polymer-based nanocomposite hydrogels. Laponite RD, a synthetic 2-D nanoclay that becomes inert when in a physiological environment, while mixed with water, becomes a clear gel with interesting shear-thinning properties. Adding Laponite RD to chitosan or gelatin allows for the modification of the mechanical properties of such materials. The setup explored in this research allows for a promising polymeric matrix that can potentially be loaded with active compounds for antibacterial support in foot ulcers, as well as enzymes for wound debridement.

## 1. Introduction

Nanotechnology, first introduced by Richard Feynman in 1959 as a new concept in science [1], was formally and experimentally introduced to the world in 1981 when IBM scientists Gerd Binnig & Heinrich Rohrer developed the first scanning tunneling microscope (STM). Such technology allowed them to see single atoms for the first time by scanning a tiny probe over the surface of a silicon crystal [2,3]. By studying the differences in surface area to volume ratio, physicochemical stability, and drug delivery capabilities at molecular to atomic levels, compared to the micro- or even the more, with the macroscale, it has been found that there are vast variations in the properties of matter in terms of mechanical strength, thermal and/or electric conductivity and surface roughness, to mention some examples. This is due to techniques such as “bottom-up” nanofabrication, which allow tunability of materials at the molecular scale [4].

**Figure 1:**
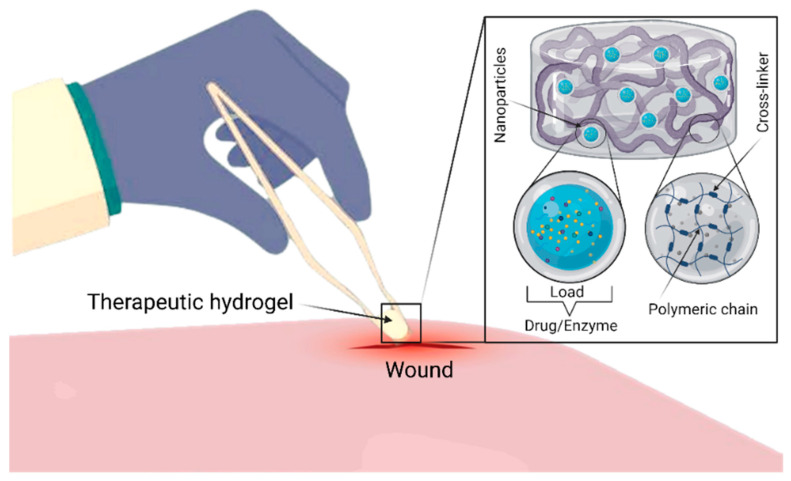
A simplified representation of polymer-based therapeutic gel deployment for wound healing. The Figure was created with “BioRender.com” template and exported under the terms of premium subscription.

Materials science is a field in which nanotechnology is being greatly explored, due to how much the bulk and surface properties previously mentioned, such as structural tunability, functionalization, and physicochemical stability, etc., are observed to change with diverse synthetization protocols in order to form customized nanostructured materials [5,6]. Materials properties, such as shape, size, crystal structure, and surface roughness, can be taken advantage of and are currently being applied to practically any area of the biomedical field, such as wound healing and drug delivery around the globe with exceedingly successful results [7]. The use of nanostructured materials in the form of nanoparticles, nanofibers, and any shape given at the nanoscale (1–100 nm), applied towards biotechnological and/or biomedical applications, such as wound healing, treatment of emerging pollutants, and drug delivery, has been exponentially growing over the past few decades [8,9,10]. Researchers, engineers, and renowned companies have become very interested in the future of applied nanosystems and are currently striving to achieve more advanced technological options by using the outstanding and often surprising properties of nanomaterials to overcome emerging challenges and solve current problems of the modern era [11]. The scope of nanostructured materials used for biotechnological and biomedical applications is very broad due to the usage of metallic nanoparticles, nanostructured biopolymers, and ceramics in the form of nanoclays [12]. Each of these classifications has its own derivations of new subclasses of materials, such as nanocomposite hydrogels and carbon-based nanostructures, which have gained an important role in the field of nanotechnology as to even be considered and named as stand-alone materials [13]. By decreasing the size of a system to a nanometric scale, its contact surface increases exponentially while its volume decreases, leading to particular physico-chemical properties that are viable for numerous biomedical applications such as wound healing [14]. Figure 1 shows a simplified representation of polymer-based therapeutic gel deployment for wound healing. The size and shape of nanostructured systems are key factors that determine their potential in wound healing processes by influencing drug/growth factor delivery efficiency, diffusion through cell membranes, and cellular/tissue reactions [15].

Following careful considerations of several complications that reacted to the wound healing process, herein, we spotlight the insight role of nanostructured polymeric hydrogels as therapeutic ventures, which is lacking in the existing literature. Therefore, herein, an effort has been made to cover this literature gap by highlighting the unique role of nanoclay/polymer-based hydrogels and enzyme-loaded nanostructures for wound healing applications. More specifically, this review discusses the nanoclay (Laponite RD) and polymer (chitosan) based nanostructured constructs and their therapeutic potentialities in facilitating the entire wound healing process. This is followed by focusing on the trypsin delivery via nanostructured polymeric materials and the role of enzymes, e.g., protease, peroxidase, and others, in topical wound healing. Finally, the concluding notes and future considerations are also given in this review.

## 2. Chitosan-Based Composite Materials

**Figure 2:**
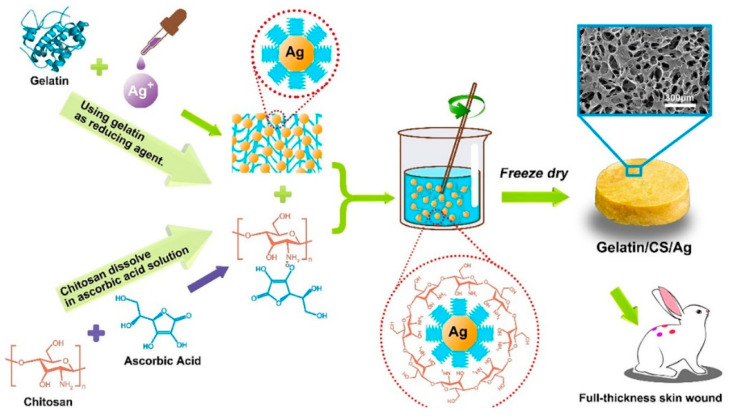
The preparation process and the principle of composite gelatin/CS/Ag-based composite with enhanced antimicrobial and wound-healing activity. Reprinted from Ref. [20] with permission from Elsevier. License Number: 5063250903839.

Due to its physicochemical properties, chitosan is a biopolymer that has drawn much attention for biomedical applications, such as drug delivery and tissue engineering. However, it is possible to improve the properties of chitosan by mixing it with other materials to obtain chitosan-based composites. Such composite materials have tunable properties that make them more efficient than using chitosan alone. One of the main disadvantages of chitosan is its poor solubility at basic pH levels [16]. Recent studies have been carried out to modify the properties of chitosan-based composite materials for a wide range of applications such as wound healing and drug delivery [17,18]. However, the poor mechanical properties of pristine hydrogel materials restrict their applications. This issue can potentially be addressed by combining the added values of different materials such as chitosan, gelatin or laponite nanoparticles within the hydrogel construct. Furthermore, the gelling features of chitosan also favorably support the development and deployment of mechanically stable hydrogels. During the reaction, the gelation process encompasses the neutralization of chitosan, which decreases the repulsive forces between positively charged groups and permits a stronger interaction of cross-linked chitosan polymeric chains [19]. The suitably cross-linked polymeric chains, in turn, support the loading of bioactive entities, such as nanoparticles, therapeutic enzymes, or drug molecules (as shown in Figure 1) for pharmaceutical and biomedical applications at large and wound dressing/healing, in particular. Additionally, the addition of nanoparticles, such as silver nanoparticles, to the polymeric matrix of chitosan has been shown to improve antibacterial properties and stability of the material, proving to be an effective alternative to treat dermal pathogens and allow for wounds to regenerate properly (Figure 2) [20].

## 3. Nanoclay-Based Materials

Nanoclays (NC) are ultra-fine (≈1 nm thick, 30 nm wide) polar nanomaterials that consist of nanoparticles, which contain materials present in bones, for example, sodium, silicates, calcium, iron, zinc, magnesium, and aluminum. Such nanoclays can successfully interact and disperse within the networks of polymeric hydrogels by virtue of their charge. It should be noted that nanoclays can be considered as promising synthetic materials as an alternative to silica nanoparticles and other ceramic materials that are used in the bio-sector due to their diverse mineral composition and their ability to combine. Among the diverse kinds of nanoclays, Laponite (Na_0.7_(Mg_5.5_Li_0.3_)Si_8_O_20_(OH)_4_) is a commercial name for a synthetic nanoclay made of nanoplatelet-shaped silicates that have strong cationic interactions which have shown great potential in the field of tissue engineering [21].

Laponite consists of high aspect ratio nanoplatelets. Due to the existence of hydroxyl groups, the nanoplatelets are charged negatively on their surface. This allows them to easily disperse at low concentrations in aqueous media. The regulatory properties of laponite towards cells have been extensively studied within gelatin (GEL), chitosan (CHI), and polyethylene glycol (PEG) matrices. Although PEG on its own has not shown an effect on cell attachment, the reinforcement of PEG with laponite stimulates the cytoskeletal arrangement of F-proteins, actin, and cell binding [22,23]. Subsequent research on nanoclay-gelatin methacryloyl (GelMa) compounds has demonstrated they have a high impact on cell proliferation and osteogenic differentiation of preosteoblasts. The compounds proved to be exceptionally strong since the elastic modulus of compression was almost four times higher when compared to pure GelMa. In addition, the fact that laponite proved to be able to promote bone mineralization without requiring the aid of growth factors was of particular interest. Therefore, hydrogels incorporated with laponite have emerged as composite materials of high interest as promising options for applications such as tissue engineering and wound healing without the necessity of growth factors [24]. Synthetic silicate nanoplatelets, such as Laponite RD, are highly charged nanoparticles that have been demonstrated to induce blood coagulation [25].

## 4. Trypsin Delivery via Nanostructured Polymeric Materials

Trypsin is well known to hydrolyze lysine and arginine residues from proteins. However, in practical applications, free trypsin suffers several problems such as high consumption, instability, and recovery difficulties from the reactor [26]. Besides, due to the self-digestion of trypsin, protein digestion in liquid media is usually inefficient and slow. To overcome these issues, enzyme immobilization is a proposed approach that leads to improved operation. This can be achieved by attaching the enzyme to reliable supports in diverse morphologies such as films, nanoparticles, nanofibers, hydrogels, etc. Amongst some of the prominent advantages of enzyme immobilization include higher enzyme stability, the easier capability of isolation from the digestion solutions for proteins, and the possibility of reusability [27].

### 4.1. Nanoparticle-Immobilized Trypsin

Immobilization methods, in which trypsin is covalently bound to the nanoparticles, can increase its stability and reusability by preventing the leakage of the trypsin [28]. Previous work by Sun et al. reported a covalent immobilization on Fe_3_O_4_ magnetic nanoparticles modified by carboxymethyl chitosan (CM-CTS) via EDC and glutaraldehyde (GA) cross-linking [29]. It was concluded that immobilized trypsin exhibited greater pH stability, which may be attributed to the conformational stabilization of the immobilized trypsin resulting from multipoint covalent cross-linking. Besides, the immobilized trypsin showed greater temperature stability than free trypsin. Another example of trypsin immobilization is given by Atacan et al., in which they covalently immobilized trypsin onto modified magnetic nanoparticles where the surface modification method improved the dispersibility, stability, and biocompatibility of the magnetic nanoparticles for specific purposes [30].

### 4.2. Nanofiber-Immobilized Trypsin

Recent works have reported different trypsin immobilization strategies such as onto electrospun nanofibers by direct covalent attachments in nonwoven nanofiber mats (NNMs) made from polystyrene/poly[styrene-co-(maleic anhydride)] mixture [31]. Silva et al. reported a very similar approach for trypsin immobilization with NNMs made of poly(ethylene terephthalate)/poly(lactic acid) copolymer. In this study, they assessed three different immobilization strategies [32], such as (a) employing a carbodiimide, direct covalent attachment immobilization; (b) immobilization through cross-linking and adsorption with glutaraldehyde; (c) covalent bonding of cross-linked trypsin agglomerates to amine-derivatized polyethylene terephthalate/polylactic acid (PET/PLA) mats. It was concluded that the lowest activity for immobilized enzymes was achieved on the PET/PLA mats due to the direct covalent bonding with the carboxylic groups [33].

## 5. Complications in Wound Healing

**Figure 3:**
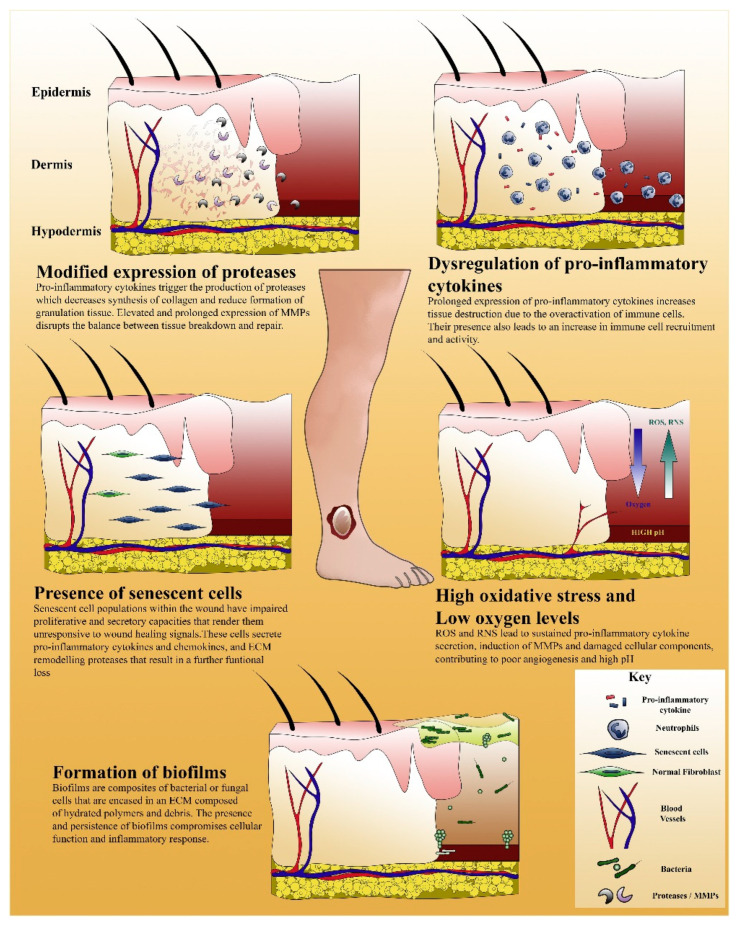
An illustration that summarizes the elements of perturbed healing found in chronic wounds. Reprinted from Ref. [37] with permission from Elsevier. License Number: 5063251014605.

Certain complications may arise during the wound healing process, which includes dehiscence, herniation, wound infection, delayed healing, and excessive scar formation [34]. A combination of overlapping factors, such as local tissue ischemia, repeated trauma and ischemia/reperfusion injury, tissue necrosis, compromised cellular and systemic stress response, and essential bacterial infection, can result in non-healing or chronic wounds [35]. Additional to the local factors, systemic ones include age, gender, hormones, stress, ischemia, diseases (diabetes, keloids, fibrosis, hereditary healing disorder, jaundice uremia), obesity, medications (glucocorticoid steroids, non-steroidal anti-inflammatory drugs, chemotherapy), alcoholism and smoking, immunocompromised conditions (cancer, radiation therapy), and nutrition [36]. Chronic wounds can generally be classified into three most common categories: diabetic foot ulcers (DFUs), pressure ulcers (PUs) and venous leg ulcers (VLUs). Given variations in molecular etiology, these wounds share similar elements: (i) modified expression of proteases, (ii) dysregulation of pro-inflammatory cytokines, (iii) presence of senescent cells, (iv) high oxidative stress and low oxygen levels, and (v) formation of biofilms [37]. In spite of the variances that exist in the molecular etiology of chronic wounds, these share common elements, as summarized in Figure 3 [37].

Moreover, a plethora of causes feed an unfavorable microenvironment that impedes cutaneous repair, such as hyperglycemia, persistent inflammation, and growth factor, and cytokine deficiencies lead to impaired stem cell recruitment for sufficient angiogenesis [38]. Therefore, an increasing interest in understanding the mechanisms that contribute to non-healing wounds, such as reduced bioavailability of growth factors and receptors, irregular production/modification of matrix proteins, decreased proliferative capacity of resident cells, and inadequate or impaired wound perfusion [39]. The previously mentioned elements shared between chronic wounds involve, in different levels, the activity of enzymes that impact negatively or positively on the wound healing process.

## 6. Role of Enzymes in Topical Wound Healing

**Figure 4:**
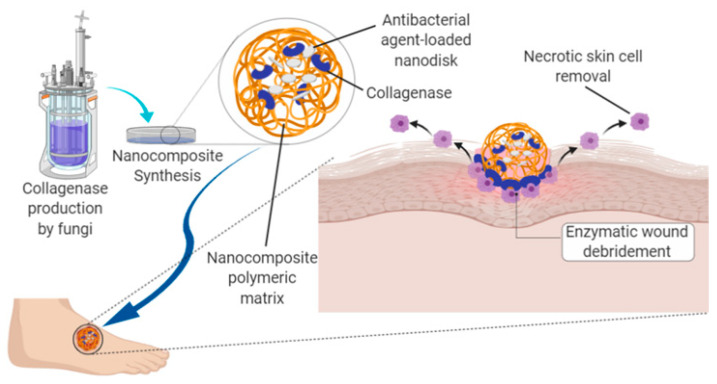
Schematic representation of the formulation of an optimized nanocomposite material by encapsulating enzymes, such as collagenase, gelatinase, or trypsin, within a polymeric matrix for treating the main setbacks in the wound healing process of diabetic foot ulcers.

Damaged tissues increase the formation of reactive oxygen species (ROS) and decrease the amount of different enzymatic and non-enzymatic free-radical collectors. The presence of ROS radicals negatively affects the wound healing process. Additionally, excessive amounts of ROS cause irritation, soaring, cell death, and minimization of the healing process [40]. Figure 4 shows the formulation of an optimized nanocomposite material by encapsulating enzymes, such as collagenase, gelatinase, or trypsin, within a polymeric matrix for treating the main setbacks in the wound healing process.

In order to avoid oxidative stress, cells have, over time, developed various systems to remove toxicity in ROS. The enzymatic strategy consists of enzymes that detoxify ROS, such as catalase, the selenium-based enzyme glutathione peroxidase (SeGPx), and superoxide dismutases (SODs). SODs catalyze the dismutation of dioxide (1-) ions into hydrogen peroxide (H_2_O_2_) and molecular oxygen (O_2_). H_2_O_2_ can be further detoxicated by glutathione (GSH) peroxidases (GPx), which include SeGPx and members of the ubiquitous peroxiredoxin (Prx) family of antioxidants [41]. Another type of enzyme that take parts in the complicated process of tissue regeneration and wound healing is proteases. It is hypothesized that very quick protease activity is present at the beginning of the healing processes in an acute wound, while in regular wounds, the enzymatic activity reaches its maximum levels in the first several days and then decreases to very low activity levels after the first week of healing progression [42]. However, in advanced wounds that are incapable of healing, it is theorized that high enzymatic activity of protease may begin through two different paths: by relating to either the bacteria within the wound or the human cells in the wound bed. Additionally, these two routes of protease activity have a synergistic mechanism [43].

**Figure 5:**
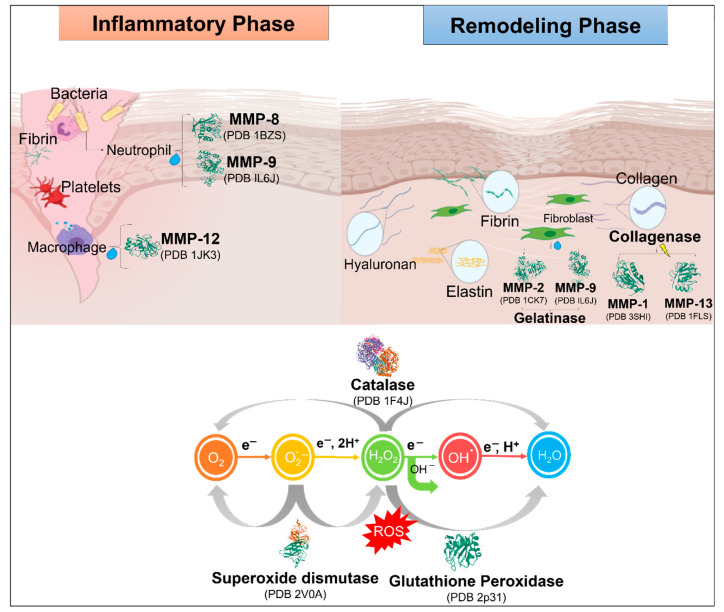
MMPs expressed by fibroblasts and inflammatory cells, such as neutrophils and macrophages, regulate the wound healing process.

During inflammation, the integration of dermal enzymes is accelerated, which then leads to the deterioration of the extracellular matrix (ECM). Some examples of this kind of enzymatic activity can be seen with elastin fibers and fibrin, which can be hydrolyzed by elastase, hyaluronidase, which depolymerizes hyaluronic acid (HA), and matrix metalloproteinases (MMPs), which have the capability of breaking type I collagen. Thus, it is suggested that the presence of dermal enzymes, as well as the downregulation of fiber formation, play a key role in the wound healing process of skin [44,45]. The downgrading and reconstruction of the ECM by proteases, particularly MMPs, is a key element of tissue reformation and is also relevant in processes such as angiogenesis, re-epithelialization and the appearance of leukocytes [46]. ECM degradation clears the path for platelets, cell growth, neutrophils, and macrophages to remove pathogens [47,48]. Neutrophils produce high levels of ROS, proteases, and pro-inflammatory cytokines to sanitize the wound. When this process is complete, apoptosis occurs on neutrophils and becomes phagocytosed by the newly arrived macrophages [49]. By secreting MMPs such as elastase or collagenase, macrophages play a role in phagocytosis and the wound debridement process, as well as eliminating bacteria [50]. Nevertheless, they are the main source of growth factors and cytokines that stimulate the fibroblast proliferation and biosynthesis of collagen. They also cause fibrin clot removal by liberating the plasminogen activator [51]. MMPs expressed by fibroblasts and inflammatory cells, such as neutrophils and macrophages, regulate the wound healing process are shown in Figure 5.

### Role of Proteases in the Wound Healing Process

Proteases and their inhibitors are key factors during the wound healing process. Proteolytic enzymes are present in different proportions during acute and chronic injuries. Proteolytic enzymes (proteases, proteinases, and peptides) are a group of proteins that help in the downgrading of necrotic skin caused by cell malfunction and/or death. These types of enzymes are often produced as precursor proteins with regulated activation. Additionally, they take part particularly in the regulation of mitosis and cell growth, synthesis of collagen and yield. They are also involved in the growth and remotion of perivascular fibrin chains, which are related to chronic venous insufficiency (CVI) and ulceration in feet and legs, as well as the removal of necrotic debris following swelling. It is a challenge to predict the outcome of applying synthetic enzymes, even if they are of the proteolytic family, to a wound, as only a limited number of enzymes are the ones that do such functions [52].

Proteases play a key role in wound treatment. They are found in severe and chronic wounds in different amounts. Equilibrium between proteases and their inhibitors is critical for the wound healing process because irregularities may result in disproportioned ECM deterioration and depositing, thus resulting in improper healing of the wound. In recent times, progress and findings in the field of healthcare have established novel methods to regulate the level of proteases, e.g., MMPs modulators which include enzyme-modulating dressing, peptides, signaling molecules, and micro-RNA [53]. The existence of proteases at high concentrations within chronic and acute wounds is the cause of ECM downgrading and reduction of cell proliferation within the wound bed. Additionally, toxins emitted by bacteria are the cause for excessive inflammation and tissue damage that can lead to cellulitis, abscess, osteomyelitis, or even amputations (e.g., in diabetic patients). Proteases have the capacity to break down antimicrobial peptides (AMPs) into functionless compounds and limit their therapeutic efficacy [54]. The following list enumerates the participation of proteolytic enzymes in wound healing processes:

✓ They are of high importance in wound management, and maintaining a balance between them prevents irregularities such as immoderate ECM degradation and depositing, thus resulting in impaired healing. An unbalanced process usually leads to abnormal scarring [55]. Furthermore, when conjugated to other complications, such as diabetes, it leads to continuous inflammation and non-healing [56].
✓ Enzymes that catalyze protein hydrolysis into minor portions/particles such as peptides. They can be grouped based on their protein substrate, optimal pH, their cut specificity (the amino acid peptide bond hydrolyzing), and their catalytic site configuration.
✓ Proteases are divided into two main categories: endopeptidases and exopeptidases.
✓ Exopeptidases focus on the N- and C-terminations of peptide bonds, while endopeptidases separate peptide linkages apart from the endpoint of the protein substrate.
✓ Based upon the active site configuration, proteases are classified as serine, aspartic, cysteine and metalloproteases.
✓ Other proteases that do not fall into the conventional classifications are the ATP-dependent proteases.
✓ A characteristic major function of proteolytic enzymes is to regulate the ratio between tissue regeneration and tissue degeneration.
✓ They have an essential role in the transfer and stimulation of fibroblasts, ECM restoration and growth factor activation.
✓ During the different stages of inflammation, they take part in damaged tissue removal (debridement) and influence bacterial load in the wound area.
✓ During the stage of cell proliferation, they are quickly found at the formation of blood vessels to facilitate vascularization during angiogenesis.
✓ In the ending phase of growth and restructuring, they absorb the ECM and assist in tissue regeneration. It has been estimated that over 100 enzymes are involved in this phase.
✓ The proteases that are involved the most in the wound healing process are MMPs and metalloproteinases of the thrombospondin domain (ADAM-TS, tolloids, serine proteases, pappalysins, and meprins [57].

## 7. Classification of Proteases Involved in the Wound Healing Process

Matrix metalloproteinases (MMPs): MMPs are a group of calcium-dependent, zinc-containing enzymes. In different tissues, they have been identified 24 different MMPs, varying substrate specifications, and multiple functions [58]. Besides, during microbial infection, the MMPs play an essential role by degrading the extracellular matrix products from different organs that exhibit antimicrobial activity against wound pathogens [59]. Based on MMPs domain organization and substrate preference, these can be classified into the four most relevant groups: (1) collagenases, (2) gelatinases, (3) stromelysins, and (4) matrilysins. Collagenase: This group is comprised of enzymes MMP-1, MMP-8, and MMP-13, which are the enzymes in mammals with the capability to break down the triple helix of collagen [60]. Such MMPs can also downgrade several other non-ECM and ECM molecules. Interstitial collagenase (MMP-1) breaks type II collagen and appears to have activity, especially with type III collagen. Polymorphonuclear collagenase (MMP-8) has the most significant activity against type I. MMP-13 has a unique intensive capability to break all three types of collagen (I, II, and III) [61]. Gelatinases: MMP-9 (gelatinase B) and MMP-2 (gelatinase A) are the main enzymes that are upregulated in chronic wounds [62]. Fibroblasts secrete MMP-2, while the larger molecule MMP-9 is mainly produced by leukocytes and, possibly, by keratinocytes. These enzymes play an essential role in the remodeling because they have additional fibronectin located inside the catalytic domain. One of the main functions of gelatinase A is to accelerate migration, while gelatinase B promotes cell migration and re-epithelialization [63]. Stromelysins: This group is composed of three members: Stromelysin-1 (MMP-3), stromelysin-2 (MMP-10), and stromelysin-3 (MMP-11), which play a varied role in the degradation of the extracellular matrix [64]. Stromelysins are expressed by epithelial and fibroblast cells and are secreted to the extracellular space, where they play essential roles in biological processes such as mammary gland development, immunity, and wound healing [65]. Matrilysins: During the process of tissue remodeling, MMP-7, also known as matrilysins-1, is believed to degrade components of the extracellular matrix (ECM) such as laminin, entactin, and type IV collagen [66]. Additionally, in humans, MMP7 expression is observed in IPF lung tissue but not healthy control samples. It is also detectable in BAL fluid, where levels are increased in patients with IPF and inversely correlated with FVC [67]. Serine proteases: Serine proteases are proteins with abundant sources distributed among all living cells and are important enzymes because some of them hydrolyze peptide bonds [68]. These proteins contain serine residues in their active catalytic center, which has a molecular mechanism similar to esterase. Serine protease derives its name from the presence of residual nucleophilic serine in the active site that attacks the carbonyl components of the substrate [69].

The enzymatic activities of serine proteases are tightly regulated within translation transcription, zymogen activation, autolysis, and interaction with natural inhibitors. Thrombin, is one of the most noticeable members of the serine protease family, is a 36*-*kDa protein comprised of two chains, A and B, linked by a disulfide bond [70]. Grouping peptidases classify proteolytic enzymes based on sequencing similarities and structure into families and clans based on catalytic mechanism, PA Clan proteases and the E*form, and homology [71].

✓ Catalytic mechanism: Enzymes that exhibit proteolytic activity are grouped as glutamic, cysteine, threonine, serine, asparagine, aspartic, or metalloproteases. Stimulation of many trypsin-like proteases of the serine group requires proteolytic processing of an idle zymogen precursor. Practically all PA Clan proteases utilize the canonical catalytic triad and hydrolyze the peptide bond via two tetrahedral intermediates [72].
✓ PA Clan Proteases: The largest family of serine proteases is the PA proteases clan that is present in the trypsin fold and is possibly the best-studied group of enzymes currently [73]. Most proteases of the PA clan have specificity for substrates similar to trypsin and prefer the Lys and/or Arg chains at the P1 position. Additionally, trypsin and chymotrypsin are known to be digestive enzymes that break polypeptide chains of positively charged or large hydrophobic residues, respectively. This type of proteases relies on several crucial biological processes such as blood clotting and immune response, which involve torrents of sequential zymogen activation [74].
✓ E*form: The critical serine protease in recent kinetic studies on thrombin showed that the blood coagulation pathway asserts for unpredictable plasticity of the trypsin fold [69]. Thrombin exists in three forms at equilibrium, such as Na^+^-free form E, Na^+^-bound form E, and E* [75]. Where Na^+^ are the low and high activity configurations of the enzyme, Na^+^-bound being the cause of the procoagulant, prothrombotic, and signaling activities. Another form, E*, is in balance with E and is idle toward the substrate and, therefore, it is unable to link Na^+^ [69].

Dead tissues present in a wound site serve as reservoirs for the development of bacteria and contain high levels of inflammatory mediators, which promote a continuous case of inflammation and reduce cellular migration that is necessary for wound regeneration. Proper wound cleaning and debridement are elemental for granulation, followed by re-epithelization. Among the known debridement methods, enzymatic debridement is a highly efficient method that uses proteolytic enzymes naturally present in the body.

While proteases are not recommended for use in delivery routes such as oral, due to their susceptibility to inhibitors, ease of denaturing, and need to remain long enough in the site of action to achieve positive pharmacokinetics, they have been successfully studied and applied via the topical route. The most frequently used proteases for topical wound healing applications include collagenases, cysteine proteases, and serine proteases, although animal secretions such as snake venom, *Lucilia sericata* secretions, and fish epithelial mucus, all of which include several enzymatic and non-enzymatic proteins (proteases being among these) have been demonstrated to have good results [76,77,78].

## 8. Conclusions

The role and importance of enzymes in the wound healing process is a topic currently being actively explored by researchers as a novel alternative for wound debridement and healing. It is a painless and quick method that excels over the traditional methods, especially for patients with chronic wounds and potential amputations. Another important concept of current interest is the capability of applying proteolytic enzymes to a wound bed through different nanomaterials such as nanoparticles, nanofibers, and hydrogels. Nanocomposite-based materials such as hydrogels, not only are able to deliver the enzymatic compound to the wound, but they may also have other roles while doing so, such as stabilizing the enzymatic activity, modifying environmental conditions of the wound site, and acting as antibacterial agents. In conclusion, the encapsulation of enzymes within nanomaterials, such as hydrogels, has become of great interest due to the number of possibilities it opens for the biomedical field.
